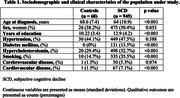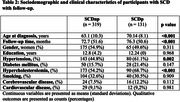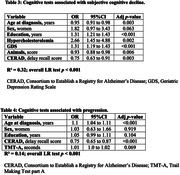# Clinical and neuropsychological variables associated with subjective cognitive decline and its progression

**DOI:** 10.1002/alz.089217

**Published:** 2025-01-03

**Authors:** Rafael Angel Villino‐Rodríguez, Mirla Ríos‐Rivera, Laura Imaz, Christian Espinoza‐Vinces, Cristina Pérez‐Prol, Genoveva Montoya‐Murillo, Carlota Arrondo, Mario Riverol

**Affiliations:** ^1^ Clinica Universidad de Navarra, Pamplona, Navarra Spain; ^2^ Clínica Universidad de Navarra, Pamplona, Navarra Spain; ^3^ Hospital Universitario de Navarra, Pamplona, navarra Spain; ^4^ University Clinic of Navarra, Pamplona, navarra Spain

## Abstract

**Background:**

This study focused on comparing clinical and neuropsychological aspects in individuals with subjective cognitive decline (SCD) versus healthy controls (HCs) in a memory clinic, aiming to identify factors linked to the progression towards mild cognitive impairment (MCI) or dementia.

**Method:**

We retrospectively analysed data from 945 SCD patients and 68 HCs at Clínica Universidad de Navarra memory clinic between 2001 and 2017, with 450 followed up until January 2020, the study involved various assessments including medical interviews, lab tests, neuropsychological evaluations (during the first interview and follow‐ups), and brain imaging. All the neuropsychological variables were adjusted for age, sex and education.

**Result:**

Among the participants with SCD, 131 progressed to MCI or dementia. SCD individuals were younger, and had higher educated, and more vascular risk factors compared to HCs. Factors associated with MCI or dementia encompassed age at diagnosis, years of education, and hypercholesterolemia. Neuropsychologically, SCD individuals exhibited weaker global cognition, verbal memory, fluency in language, and increased depressive symptoms. Poorer performance in episodic verbal memory tests correlated with a higher likelihood of transitioning to MCI or dementia.

**Conclusion:**

The study’s discussion emphasized the complex nature of SCD development and progression, involving both clinical and neuropsychological factors. Younger age and increased vascular risk were characteristic of SCD individuals, who also showed deficiencies in global cognition, language fluency, verbal memory, and executive functions. Key indicators of disease progression included age at diagnosis, hypercholesterolemia, and poorer performance in tests assessing language fluency, verbal and visual memory, and executive functions.